# The association between chronic heart failure and frailty index: A study based on the National Health and Nutrition Examination Survey from 1999 to 2018

**DOI:** 10.3389/fcvm.2022.1057587

**Published:** 2023-01-09

**Authors:** Xiaozhe Chen, Chunlei Hou, Lei Yao, Yulong Ma, Yunfeng Li, Jianhua Li, Mingtai Gui, Mingzhu Wang, Xunjie Zhou, Bo Lu, Deyu Fu

**Affiliations:** Department of Cardiology, Yueyang Hospital of Integrated Traditional Chinese and Western Medicine, Shanghai University of Traditional Chinese Medicine, Shanghai, China

**Keywords:** chronic heart failure, frailty, National Health and Nutrition Examination Survey, cardiovascular and cerebrovascular disease, logistic regression

## Abstract

**Objective:**

This study aims to explore the association between the frailty index and chronic heart failure (CHF).

**Methods:**

We collected data from the National Health and Nutrition Examination Survey (NHANES) (1998–2018) database to assess the association between CHF and frailty. Demographic, inquiry, laboratory examinations, and characteristics were gathered to compare CHF and non-CHF groups. Multiple logistic regression analysis was performed to explore the relationship between frailty and CHF. Cox proportional hazards models were used to estimate hazard ratios (HR) and 95% confidence interval (CI) for mortality from all causes and cardiovascular disease (CVD).

**Results:**

A total of 16,175 participants with cardiac and cerebrovascular disease were categorized into CHF (*n* = 1,125) and non-CHF (*n* = 15,050) groups. In patients with CHF, the prevalence of frailty, pre-frailty, and non-frailty were 66.31, 30.93, and 2.75%, respectively. In multiple logistic regression, patients with CHF who were male (OR = 0.63, 95% CI: 3.11–5.22), whose annual family income was over $20,000 (OR = 0.52, 95% CI: 0.37–0.72, *p* < 0.001), or with normal hemoglobin level (OR = 0.77, 95% CI: 0.68–0.88, *P* < 0.001) had a lower likelihood of frailty. CHF patients with hypertension (OR = 3.60, 95% CI: 2.17–5.99, *P* < 0.0001), coronary heart disease (OR = 1.76, 95% CI: 1.10–2.84, *P* = 0.02), diabetes mellitus (OR = 1.89, 95% CI: 1.28–2.78, *P* < 0.001), and stroke (OR = 2.52, 95% CI: 1.53–4.15, *P* < 0.001) tended to be frail. Survival analysis suggested that pre-frailty and frailty were related to poor all-cause deaths (HR = 1.48, 95% CI: 1.36–1.66; HR = 2.77, 95% CI: 2.40–3.18) and CVD mortality (HR = 1.58, 95% CI: 1.26–1.97; HR = 2.55, 95% CI: 2.02–3.21). CHF patients with frailty were strongly connected with all-cause death (HR = 2.14, 95% CI: 1.27–3.62).

**Conclusion:**

Frailty was positively associated with CHF. Patients with CHF who were male, whose annual family income was over $20,000, or with normal hemoglobin level were negatively correlated to frailty. For patients with cardiac and cerebrovascular disease as well as CHF, frailty was strongly connected with all-cause death.

## 1. Introduction

Chronic heart failure (CHF) is one of the leading causes of death globally. It is a complex clinical syndrome with symptoms and signs that result from any structural or functional impairment of ventricular filling or ejection of blood (1). The prevalence of adult CHF accounts for ~1–2% in developed countries, which is expected to rise to 3% by 2030 worldwide (2). In the United States, about 6.2 million individuals are suffering from CHF (3). On current trends, the costs of treatment are expected to rise from $20.9 billion to $53.1 by 2030.

It was reported that the prevalence of patients with CHF was about 30–90% (4, 5). Frailty could predict all-cause death rates and CHF-related hospitalization according to other studies (6). However, a standard definition of frailty remains lacking, but there exists a recognized definition provided by the World Health Organization (WHO), that is, a clinically recognizable state in which the ability to cope with everyday or acute stressors is compromised by an increased vulnerability brought by an age-associated decline in physiological reserve and function across multiple organ systems (7). As a syndrome with a serious reduction of several physiological system functions, frailty could lead to a lower homeostatic tolerance of stressors. The decline of various body functions will rise the sensitivity and vulnerability to adverse factors (8). The Cumulative Deficit Model developed by Rockwood et al. (9) is one of the most used measurements of frailty. It is based on a comprehensive geriatric assessment and combines four parts, including chronic conditions, psychosocial factors, cognitive deficits, and other geriatric signs and symptoms.

To the best of our knowledge, most studies focusing on CHF and frailty were prospective or retrospective studies, which had limited sample size and follow-up time. There are no articles on CHF and frailty in the online databases. The National Health and Nutrition Examination Surveys (NHANES) is a well-designed large-sample clinical registration database with a complete follow-up, which can well discuss the correlation between frailty index (FI) and CHF and the prognosis of CHF patients with frailty. Therefore, we used the NHANES database from 1998 to 2018 to study the relationship between frailty and CHF to supplement the clinical studies.

## 2. Methods

### 2.1. NHANES database

Data were collected from the NHANES database, which is a cross-sectional survey among all non-institutionalized civilians in the United States. Trained interviewers and examinations collected data self-reported from participants using the Computer-Assisted Personal Interview system. NHANES data are published on a 2-year cycle. To obtain a large sample for analysis, a total of 10 cycles of data from 1999 to 2018 were included in this study. More details about NHANES are available at http://www.cdc.gov/nhanes.

### 2.2. Disease diagnosis and scale assessment

#### 2.2.1. Diagnosis of disease

CHF, Coronary Heart Disease (CHD), angina, stroke, and heart attack were diagnosed based on the Monetary Choice Questionnaire (MCQ) by asking “Someone ever told you had congestive heart failure (or CHD/angina/stroke/heart attack)?”

The diagnosis criteria for hypertension were as follows: (1) the answers to the MCQ questionnaire; (2) the patient's blood pressure; and (3) whether the patient is taking antihypertensive medicine; if the patient meets one of the above conditions, then the diagnosis of hypertension can be made.

The diagnosis criteria for diabetes (DM) were as follows: (1) the answers to the MCQ questionnaire; (2) glycohemoglobin (%) > 6.5; (3) fasting glucose (mmol/l) ≥ 7.0; (4) random blood glucose (mmol/l) ≥ 11.1; (5) 2-h OGTT blood glucose (mmol) ≥ 11.1; and (6) use of diabetes medication or insulin. If the patient meets one of the above conditions, then the diagnosis of DM can be made.

The diagnosis criteria of the prediabetic (preDM) phase were as follows: (1) patients who answered “yes” to the MCQ questionnaire; (2) glycohemoglobin (%): 5.7–6.5 mmol/L; (3) fasting glucose (mmol/l): 5.6–7.0; and (4) 2-h OGTT blood glucose (mmol): 7.8–11. If the patient meets one of the above conditions, then the diagnosis of prediabetic can be made.

#### 2.2.2. FI calculation

The details of the FI are listed in Supplementary Table S1 based on the study of Hakeem et al. (10). The FI included 53 deficits covering cognition, dependence, depression, comorbidities, hospital utilization, general health, physical performance, body mass index (BMI), and laboratory values. The FI is calculated by the number of acquired deficits divided by the total counts of potential deficits. Frailty is diagnosed when the FI is more than 0.25, pre-frailty when the FI is between 0.16 and 0.25, and non-frailty when the FI is < 0.16.

### 2.3. Study population

**Figure 1:**
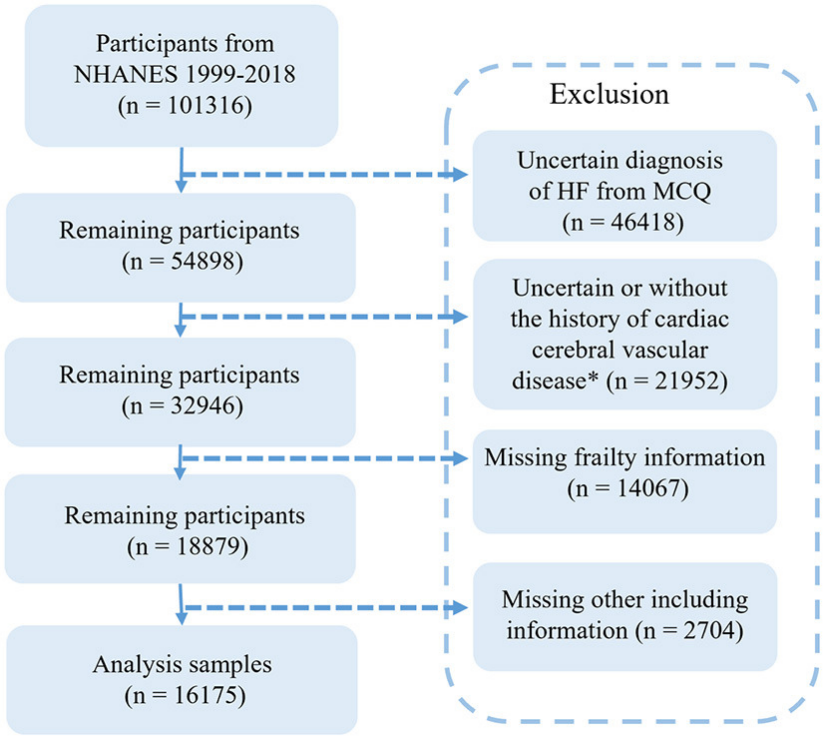
Flow chart of the screening process for the selection of eligible participants in NHANES 1999–2018.

The detailed data extraction process from the NHANES is shown in Figure 1. We extracted data from a 10-year circle from the NHANES database. There were 101,316 participants, of which 46,418 patients had an unclear diagnosis of CHF. To reduce confounding factors, a control group was set as patients with at least one of the basic diseases, including hypertension, DM, preDM, angina, stroke, and CHD. Therefore, another 21,952 participants were excluded. Then, we collected frailty information from the NHANES database. The frailty score was extracted based on the standard procedure containing 53 questions (10). To include more participants, we added participants completing 80% of the content of the questionnaire and excluded 14,067 respondents with serious deficiencies in the frailty scale (entry completion < 42), in addition to 2,704 respondents with incomplete basic information data (including blood counts, BMI, blood pressure, education, and income). Finally, a total of 16,175 patients were included.

### 2.4. Data analysis

We used R (4.2.0) and R studio for data analysis. The weighting parameters were based on the weights provided internally by NHANES mainly. Weighting was performed using wtmec4yr when the data years were 1999–2000 and 2001–2002, and wtmec2yr when the data were for the remaining years. Continuous data were described by means and standard errors with categorical comparisons, using weighted *t*-tests. We used the Chi-square test to analyze categorical variables according to the number of cases and weighted prevalence [*n* (weighted %)]. The relationship between frailty and CHF was explored by the multiple logistic regression analysis. Cox proportional hazards models were used to estimate hazard ratios (HR) and 95% confidence interval (CI) for mortality from all causes and cardiovascular disease (CVD).

## 3. Results

### 3.1. Characteristics of participants

Altogether 16,175 participants [mean age (±SE) 63.72 ± 0.21] were included in our analysis, which represented 85,222,240 adults in the United States. The mean BMI of participants was 30.00 ± 0.09 kg/m^2^, with a relatively balanced gender distribution; 15,050 were non-CHF participants while 1,125 were CHF participants. In terms of race, non-Hispanic white people accounted for 50.55% (*n* = 8,177), followed by non-Hispanic black people (*n* = 3,386, 20.93%), Mexican-Americans (*n* = 2,286, 14.13%), other Hispanic races (*n* = 1,243, 7.68%), and other races (*n* = 1,083, 6.07%). The percentage of patients with frailty was 21.64% and pre-frailty was 48.04%. For patients with a CVD history, hypertension accounted for 78.55% (*n* = 12,706), followed by preDM (*n* = 6,189, 38.26%), DM (*n* = 5,402, 33.40%), heart attack (*n* = 1,594, 9.85%), CHD (*n* = 1,572, 9.72%), stroke (*n* = 1,391, 8.60%), and angina (*n* = 1,044, 6.45%).

### 3.2. Characteristics of participants with CHF

**Figure 2:**
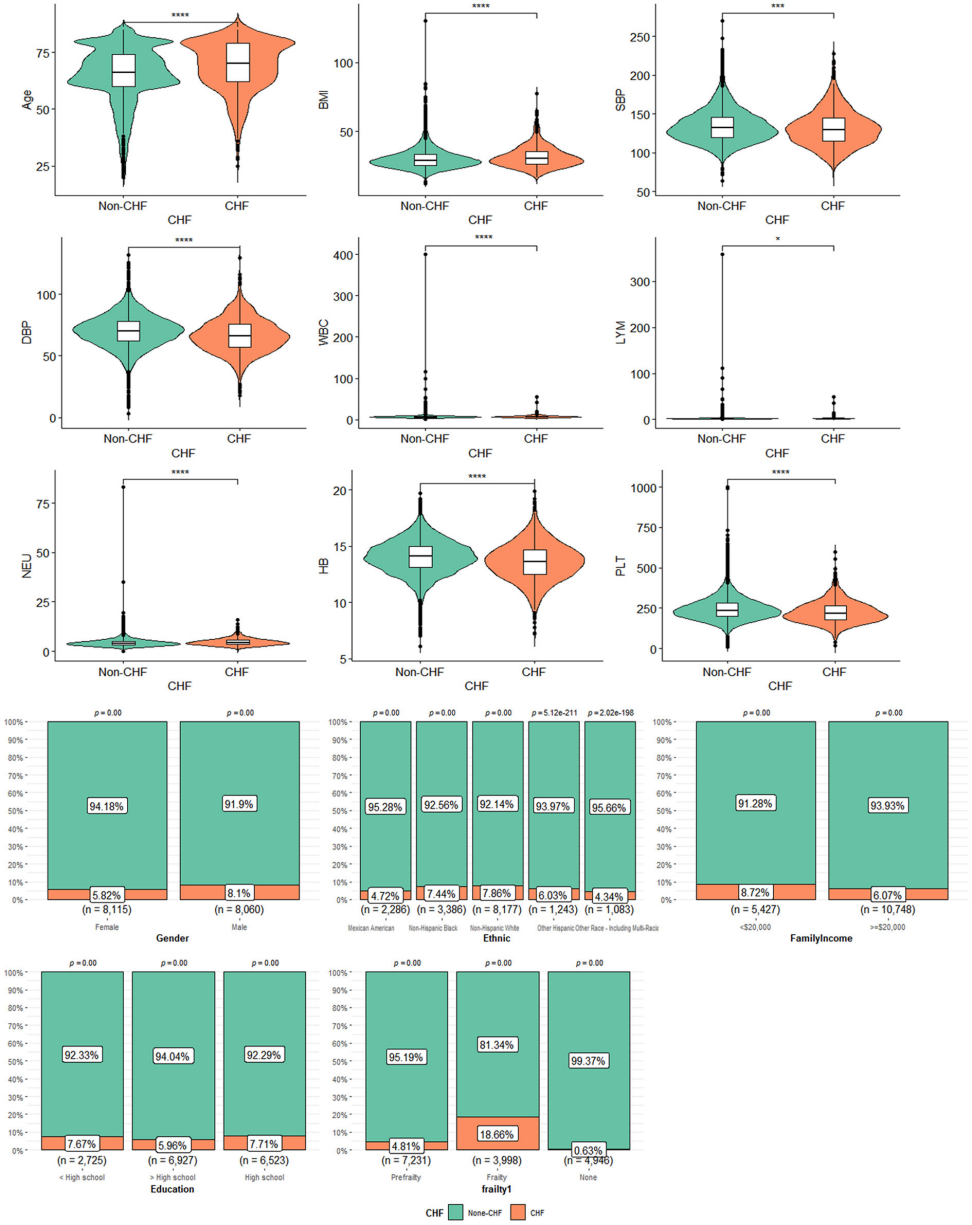
The characteristics of participants with or without CHF: *means *P* < 0.05; ***means *P* < 0.001; ****means *P* < 0.0001.

The basic information of CHF participants is shown in Table 1. Altogether 1,125 participants were CHF, representing 5,310,778 CHF adults in the United States. Patients with CHF were elder [mean (±SE) 68.03 ± 0.59 vs. 63.44 ± 0.21 years, *P* < 0.001], with a higher BMI [mean (±SE) 31.59 ± 0.38 vs. 29.89 ± 0.09 kg/m^2^, *P* < 0.001], a lower annual family income (< $20,000, CHF = 473, 39.62% vs. non-CHF = 4,954, 26.59%, *P* < 0.001), and a lower percentage of education (>High school, CHF = 413, 38.06% vs. non-CHF = 6,514, 50.09%, *P* < 0.001) than those without CHF. In terms of CVD history, patients with CHF had a higher ratio of hypertension, DM, angina, stroke, and CHD. It was an important phenomenon that patients with CHF presented a higher percentage of frailty than patients with non-CHF. In a routine blood test, patients with CHF had a combination of higher white blood count (WBC) (7.68 ± 0.09 vs. 7.31 ± 0.03, *P* < 0.0001) and neutrophilic granulocyte (Neu) (4.76 ± 0.06 vs. 4.37 ± 0.02, *P* < 0.0001), and lower hemoglobin (Hb) (13.80 ± 0.08 vs. 14.23 ± 0.03, *P* < 0.0001) and platelets (Plt, 231.23 ± 3.24 vs. 249.50 ± 0.98, *P* < 0.0001). In blood pressure, patients with CHF had lower blood pressure than patients with non-CHF for systolic blood pressure (SBP, 129.95 ± 0.89 vs. 133.33 ± 0.32, *P* < 0.01) and diastolic blood pressure (DBP, 66.55 ± 0.70 vs. 70.90 ± 0.21, *P* < 0.0001). There were non-significant differences in race, gender, and lymphocyte (Lym) percentage between patients with CHF and non-CHF. The data can be visualized in Figure 2, in which the variables of age, BMI, and Hb showed significant differences.

**Table 1:** Characteristics of participants with and without CHF.

**Characteristic**	**Total**	**CHF**	**Non-CHF**	***P*-value**
*N*	16,175	1,125	15,050	
Age, years, Mean ± SD	63.72 ± 0.21	68.03 ± 0.59	63.44 ± 0.21	< 0.0001
Gender, *N* (%)				0.01
Female	8,115 (50.17)	472 (48.26)	7,643 (53.67)	
Male	8,060 (49.83)	653 (51.74)	7,407 (46.33)	
Race, *N* (%)				0.04
Mexican American	2,286 (14.13)	108 (3.23)	2,178 (4.43)	
Non-hispanic black	3,386 (20.93)	252 (11.38)	3,134 (9.96)	
Non-hispanic white	8,177 (50.55)	643 (77.39)	7,534 (75.98)	
Other hispanic	1,243 (7.68)	75 (4.57)	1,168 (4.29)	
Other race or multi-racial	1,083 (6.70)	47 (3.43)	1,036 (5.35)	
Annual family income, *N* (%)				< 0.0001
< $20,000	5,427 (33.55)	473 (39.62)	4,954 (26.59)	
≥$20,000	10,748 (66.45)	652 (60.38)	10,096 (73.41)	
Education, *N* (%)				< 0.0001
< High school	2,725 (16.85)	209 (14.32)	2,516 (9.08)	
>High school	6,927 (42.83)	413 (38.06)	6,514 (50.09)	
High school	6,523 (40.33)	503 (47.62)	6,020 (40.83)	
BMI				< 0.001
≤ 20	509 (3.15)	35 (2.77)	474 (3.20)	
20–25	3,161 (19.54)	174 (15.36)	2,987 (19.41)	
25–30	5,645 (34.90)	341 (30.75)	5,304 (35.05)	
>30	6,860 (42.41)	575 (51.11)	6,285 (42.35)	
BMI, kg/m^2^, Mean ± SD	30.00 ± 0.09	31.59 ± 0.38	29.89 ± 0.09	< 0.0001
**Cardiovascular diseases**, ***N*** **(%)**				
Hypertension	12,706 (78.55)	988 (88.79)	11,718 (77.63)	< 0.0001
DM	5,402 (33.40)	558 (42.75)	4,844 (25.95)	< 0.0001
Angina	1,044 (6.45)	302 (31.86)	742 (5.60)	< 0.0001
Heart attack	1,594 (9.85)	519 (46.42)	1,075 (7.03)	< 0.0001
Stroke	1,391 (8.60)	236 (20.45)	1,155 (6.77)	< 0.0001
PreDM	6,189 (38.26)	309 (29.88)	5,880 (39.53)	< 0.0001
CHD	1,572 (9.72)	497 (44.29)	1,075 (7.54)	< 0.0001
Frailty, *N* (%)				< 0.0001
Frailty	3,998 (26.56)	746 (64.10)	3,252 (18.82)	
Pre-frailty	7,231 (48.04)	348 (33.22)	6,883 (45.93)	
None	4,946 (32.86)	31 (2.67)	4,915 (35.25)	
WBC, 10^3^/μl, Mean ± SD	7.33 ± 0.03	7.68 ± 0.09	7.31 ± 0.03	< 0.0001
Lym, %, Mean ± SD	2.08 ± 0.02	2.00 ± 0.05	2.09 ± 0.02	0.08
Neu, 10^3^/μl, Mean ± SD	4.40 ± 0.02	4.76 ± 0.06	4.37 ± 0.02	< 0.0001
Hb, g·dl, Mean ± SD	14.20 ± 0.03	13.80 ± 0.08	14.23 ± 0.03	< 0.0001
PLT,10^3^/μl, Mean ± SD	248.36 ± 0.98	231.23 ± 3.24	249.50 ± 0.98	< 0.0001
SBP, mmHg, Mean ± SD	133.12 ± 0.30	129.95 ± 0.89	133.33 ± 0.32	< 0.001
DBP, mmHg, Mean ± SD	70.63 ± 0.22	66.55 ± 0.70	70.90 ± 0.21	< 0.0001

### 3.3. Characteristics of participants with frailty

**Figure 3:**
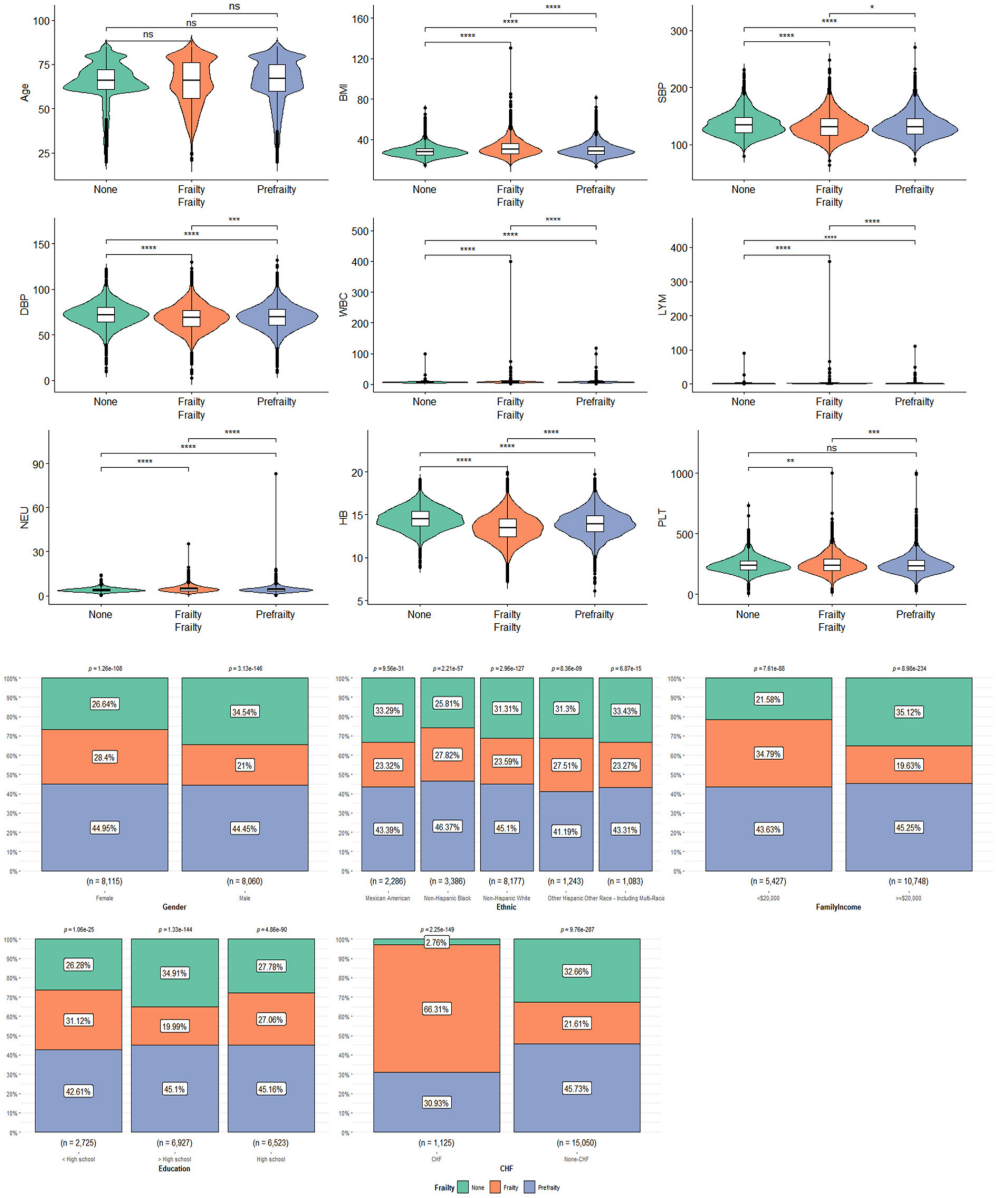
The characteristics of participants with frailty, pre-frailty, and non-frailty: *means *P* < 0.05; **means *P* < 0.01; ***means *P* < 0.001; ****means *P* < 0.0001.

Table 2 shows the characteristics of participants with frailty, pre-frailty, and non-frailty (24.70 vs. 44.70 vs. 30.57%). Our study showed no significant difference in age among the three groups of patients. Female participants tended to have frailty (62.17 vs. 37.83%, *P* < 0.01) and pre-frailty (54.27 vs. 45.73%, *P* < 0.01). Non-Hispanic white people had a lower percentage of frailty than pre-frailty and non-frailty while the rest of the races had the highest percentage of frailty in the research. Patients with frailty showed higher BMI compared to patients with pre-frailty and non-frailty [mean (±SE) 31.96 ± 0.18 vs. 30.19 ± 0.12 vs. 28.46 ± 0.13, *P* < 0.01)] significantly. A greater proportion of patients with frailty were suffering from CVD and other chronic diseases, including CHF, hypertension (85.52 vs. 80.54, 70.61%, *P* < 0.0001), DM (42.73 vs. 30.03 vs. 12.61%, *P* < 0.0001), angina (16.97 vs. 7.07 vs. 1.11%, *P* < 0.0001), heart attack (19.19 vs. 9.97 vs. 2.52%, *P* < 0.0001), stroke (17.77 vs. 6.91 vs. 1.99%, *P* < 0.0001), and CHD (19.92 vs. 10.22 vs. 2.72%, *P* < 0.0001), than patients with pre-frailty and non-frailty. There were more non-frailty patients with preDM than patients with frailty and pre-frailty (46.27 vs. 31.29 vs. 37.18%, *P* < 0.0001). Patients with frailty were higher in WBC (7.90 ± 0.08 vs. 7.37 ± 0.04 vs. 6.92 ± 0.04, *P* < 0.0001), Lym (2.23 ± 0.06 vs. 2.07 ± 0.02 vs. 2.00 ± 0.02, *P* < 0.0001), and Neu (4.80 ± 0.04 vs. 4.43 ± 0.03 vs. 4.10 ± 0.03, *P* < 0.0001) counts and lower in Hb (13.70 ± 0.05 vs. 14.13 ± 0.04 vs. 14.62 ± 0.03, *P* < 0.0001) count. Regarding blood pressure, patients with frailty had lower SBP [mean (±SE) 131.86 ± 0.50 vs. 132.72 ± 0.38 vs. 134.49 ± 0.43, *P* < 0.0001] and DBP (69.04 ± 0.31 vs. 69.98 ± 0.28 vs. 72.55 ± 0.30, *P* < 0.0001). The data can be visualized in Figure 3, in which the variables of age, BMI, and Hb showed significant differences.

**Table 2:** Characteristics of participants with frailty, pre-frailty, and non-frailty.

**Characteristic**	**Frailty**	**Pre-frailty**	**None**	***P*-value**
*N*	3,998	7,231	4,946	
Age, years, Mean ± SD	63.36 ± 0.36	63.88 ± 0.31	63.75 ± 0.28	0.35
Gender, *N* (%)				< 0.0001
Female	2,305 (62.17)	3,648 (54.27)	2,162 (46.29)	
Male	1,693 (37.83)	3,583 (45.73)	2,784 (53.71)	
Race, *N* (%)				< 0.0001
Mexican American	533 (4.88)	992 (4.36)	761 (4.00)	
Non-hispanic black	942 (13.81)	1570 (10.42)	874 (7.10)	
Non-hispanic white	1,929 (69.42)	3,688 (75.81)	2,560 (80.74)	
Other hispanic	342 (5.69)	512 (3.97)	389 (3.87)	
Other races including multi-racial	252 (6.21)	469 (5.44)	362 (4.30)	
Annual family income, *N* (%)				< 0.0001
< $20,000	1,888 (42.76)	2,368 (27.07)	1,171 (17.84)	
≥$20,000	2,110 (57.24)	4,863 (72.93)	3,775 (82.16)	
Education, *N* (%)				< 0.0001
< High school	848 (13.77)	1161 (9.40)	716 (6.57)	
>High school	1,385 (38.48)	3,124 (49.88)	2,418 (55.69)	
High school	1,765 (47.75)	2,946 (40.72)	1,812 (37.74)	
BMI				< 0.0001
≤ 20	136 (3.23)	202 (3.10)	171 (3.22)	
20–25	563 (13.63)	1,410 (19.04)	1,188 (22.92)	
25–30	1,162 (30.12)	2,571 (33.51)	1,912 (39.55)	
>30	2,137 (53.03)	3,048 (44.35)	1,675 (34.31)	
BMI, kg/m^2^, Mean ± SD	31.96 ± 0.18	30.19 ± 0.12	28.46 ± 0.13	< 0.0001
**Cardiovascular diseases**, ***N*** **(%)**				
CHF	746 (18.46)	348 (4.59)	31 (0.50)	< 0.0001
Hypertension	3,404 (85.52)	5,855 (80.54)	3,447 (70.61)	< 0.0001
DM	1,970 (42.73)	2,573 (30.03)	859 (12.61)	< 0.0001
Angina	578 (16.97)	420 (7.07)	46 (1.11)	< 0.0001
Heart attack	787 (19.19)	697 (9.97)	110 (2.52)	< 0.0001
Stroke	750 (17.77)	546 (6.91)	95 (1.99)	< 0.0001
PreDM	1,160 (31.29)	2,710 (37.18)	2,319 (46.27)	< 0.0001
CHD	766 (19.92)	694 (10.22)	112 (2.72)	< 0.0001
WBC, 10^3^/μl, Mean ± SD	7.90 ± 0.08	7.37 ± 0.04	6.92 ± 0.04	< 0.0001
Lym, %, Mean ± SD	2.23 ± 0.06	2.07 ± 0.02	2.00 ± 0.02	< 0.0001
Neu, 10^3^/μl, Mean ± SD	4.80 ± 0.04	4.43 ± 0.03	4.10 ± 0.03	< 0.0001
Hb, g·dl, Mean ± SD	13.70 ± 0.05	14.13 ± 0.04	14.62 ± 0.03	< 0.0001
Plt, 10^3^/μl, Mean ± SD	253.43 ± 1.99	246.99 ± 1.38	246.93 ± 1.42	0.01
SBP, mmHg, Mean ± SD	131.86 ± 0.50	132.72 ± 0.38	134.49 ± 0.43	< 0.0001
DBP, mmHg, Mean ± SD	69.04 ± 0.31	69.98 ± 0.28	72.55 ± 0.30	< 0.0001

### 3.4. Co-linearity analysis

The co-linearity study was conducted to eliminate possible co-linear relationships between different factors. It indicates that there is significant covariance between the factors when the variance inflation factor (VIF) value is over 5. This situation is prone to logistic regression bias. Thus, some factors need to be excluded based on experience. It was suggested that there was covariance between FI and frailty (VIF values of 5.59 and 6.875, respectively), and WBC, Lym, and Neu (VIF values of 185.69, 122.808, and 41.802, respectively) are the results of the covariance analysis of the factors associated with CHF. After removing FI, Lym, and Neu, the VIF values between the variables were < 5 and further regression analysis could be performed (Supplementary Table S2).

### 3.5. The association between CHF and frailty

**Figure 4:**
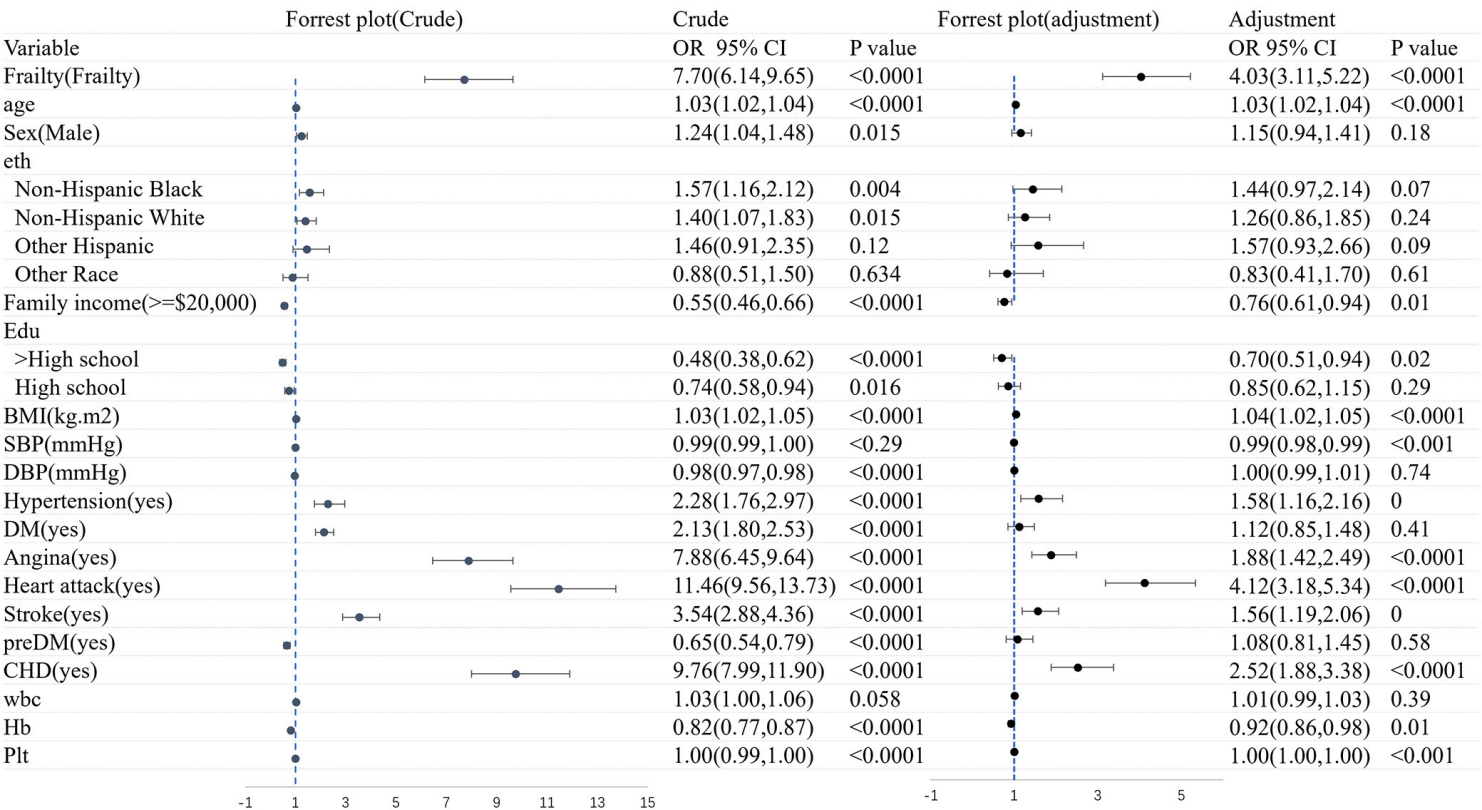
The logistic regression analysis of CHF before and after adjustments for the covariates.

Figure 4 shows the logistic regression analysis of CHF before and after adjustments for the covariates. Overall, CHF was dramatically associated with frailty (OR = 7.70, 95% CI: 6.14–9.65). There still existed an association between frailty and the morbidity of CHF (OR = 4.03, 95% CI: 3.11–5.22), after adjusting the variates of age, gender, ethnicity, family income, education, BMI, diseases, and blood test.

**Figure 5:**
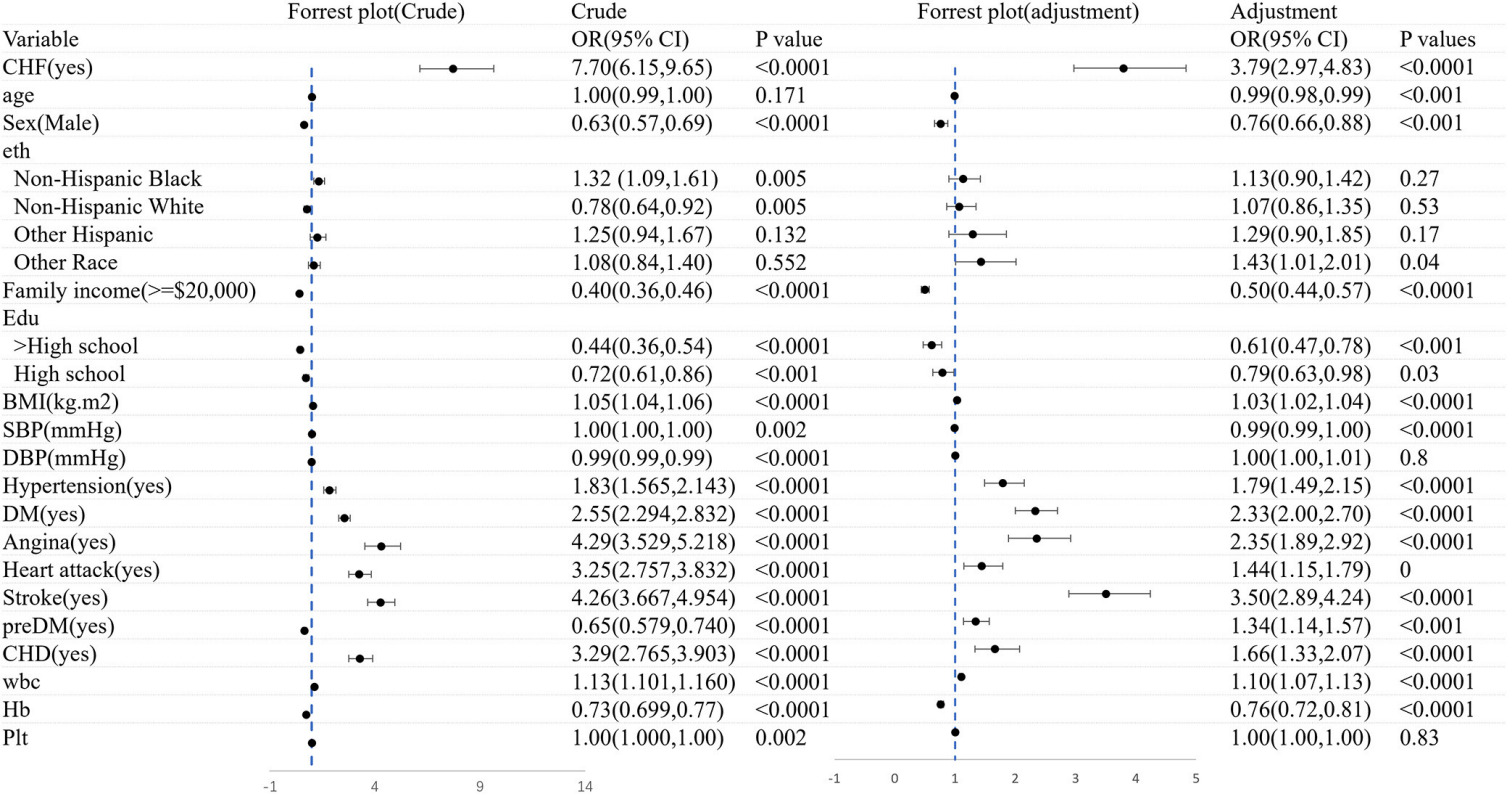
The logistic regression analysis of frailty before and after adjustments for the covariates.

Figure 5 shows the logistic regression analysis of frailty before and after adjustments for the covariates. All in all, there was a significant correlation between CHF and frailty (OR = 7.70, 95% CI: 6.15–9.65). A significant association still existed in frailty and the morbidity of CHF (OR = 3.79, 95% CI: 2.97–4.83), after adjusting for age, sex, ethnicity, family income, education, BMI, diseases, and blood test. The association was slightly weakened in the logistic regression analysis of both CHF and frailty when the covariates were adjusted.

### 3.6. Subgroup analysis

#### 3.6.1. CHF patients with frailty

**Figure 6:**
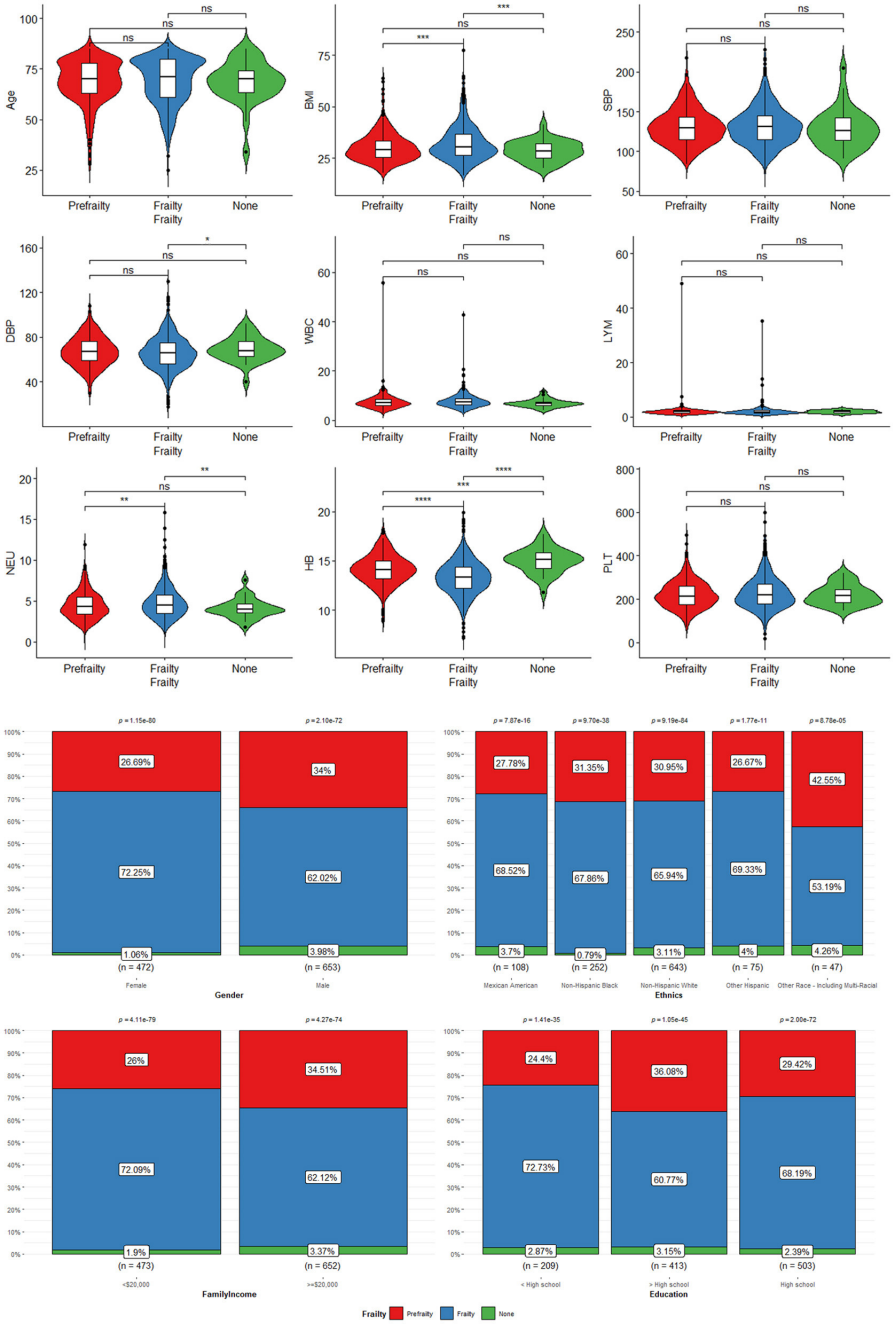
The characteristics of CHF participants with frailty, pre-frailty, and non-frailty: *means *P* < 0.05; **means *P* < 0.01; ***means *P* < 0.001; ****means *P* < 0.0001. “ns” means no statistical significance.

Table 3 shows the characteristics of CHF participants with frailty (*n* = 66.31%), pre-frailty (*n* = 30.93%), and non-frailty (*n* = 2.75%). There was no significant difference in age, race, education, prevalence of heart attack, and preDM, Plt, or SBP among the three groups of patients (*P* > 0.05). Female participants had a higher probability of frailty (55.58 vs. 44.42%, *P* < 0.0001). However, male participants were more likely to have pre-frailty (36.80 vs. 63.20%, *P* < 0.0001). Patients with CHF with annual family income < $20,000 were much easier to be frail instead of normal (45.09 vs. 12.53%, *P* < 0.0001). While those patients with an annual family income of more than $20,000 tended to be normal instead of being frail (87.47 vs. 54.91%, *P* < 0.0001). BMI was significantly higher in patients with frailty compared to patients with pre-frailty and non-frailty (32.31 ± 0.47 vs. 30.37 ± 0.50 vs. 29.46 ± 0.77, *P* < 0.001). A greater proportion of patients with frailty were suffering from cardiac and cerebrovascular disease and other chronic diseases, including hypertension (93.59 vs. 73.65%, *P* < 0.001), DM (48.21 vs. 2.13%, *P* < 0.001), angina (35.46 vs. 1.45%, *P* < 0.01), stroke (25.28 vs. 1.37%, *P* < 0.001), and CHD (47.93 vs. 14.61%, *P* < 0.001). In terms of a blood test, patients with frailty were lower in Hb (13.48 ± 0.10 vs. 15.05 ± 0.21, *P* < 0.001) and higher in Plt (236.34 ± 4.43 vs. 220.09 ± 13.33, *P* < 0.001) and DBP (65.62 ± 0.88 vs. 71.97 ± 2.40, *P* < 0.001). The data can be visualized in Figure 6, in which the variables of age, BMI, blood pressure, Neu, Plt, and Hb showed significant differences.

**Table 3:** Characteristics of CHF participants with frailty, pre-frailty, and non-frailty.

**Characteristic**	**Frailty**	**Pre-frailty**	**None**	***P*-value**
*N*	746 (66.31)	348 (30.93)	31 (2.75)	
Age, Mean ± SD	68.51 ± 0.78	67.14 ± 0.99	67.44 ± 2.24	0.53
Gender, *N* (%)				< 0.0001
Female	341 (55.58)	126 (36.80)	5 (15.38)	
Male	405 (44.42)	222 (63.20)	26 (84.62)	
Race, *N* (%)				0.25
Mexican American	74 (3.39)	30 (2.80)	4 (4.67)	
Non-hispanic black	171 (12.64)	79 (9.63)	2 (2.76)	
Non-hispanic white	424 (76.63)	199 (78.04)	20 (87.62)	
Other hispanic	52 (4.82)	20 (4.25)	3 (2.58)	
Other race	25 (2.51)	20 (5.28)	2 (2.37)	
Annual family income, *N* (%)				< 0.0001
< $20,000	341 (45.09)	123 (31.27)	9 (12.53)	
≥$20,000	405 (54.91)	225 (68.73)	22 (87.47)	
Education, *N* (%)				0.13
< High school	152 (16.30)	51 (11.11)	6 (6.65)	
>High school	251 (34.98)	149 (42.75)	13 (53.55)	
High school	343 (48.72)	148 (46.14)	12 (39.80)	
BMI, *N* (%)				0.65
≤ 20	25 (2.91)	10 (2.73)	0 (0.00)	
>30	403 (53.29)	159 (46.84)	13 (52.08)	
20–25	101 (13.65)	65 (18.64)	8 (15.86)	
25–30	217 (30.16)	114 (31.79)	10 (32.06)	
BMI, kg/m^2^	32.31 ± 0.47	30.37 ± 0.50	29.46 ± 0.77	< 0.001
**Cardiovascular diseases**, ***N*** **(%)**				
Hypertension	683 (93.59)	282 (80.74)	23 (73.65)	< 0.0001
DM	422 (48.21)	135 (35.50)	1 (2.13)	< 0.0001
Angina	231 (35.46)	70 (27.37)	1 (1.45)	0.005
Heart attack	357 (45.83)	153 (48.32)	9 (37.10)	0.65
Stroke	196 (25.28)	38 (12.66)	2 (1.37)	< 0.001
PreDM	182 (28.40)	114 (30.97)	13 (51.78)	0.09
CHD	361 (47.93)	131 (39.66)	5 (14.61)	0.01
WBC, 10^3^/μl, Mean ± SD	7.76 ± 0.13	7.56 ± 0.18	7.33 ± 0.35	0.44
Lym, %, Mean ± SD	1.98 ± 0.06	2.02 ± 0.10	2.13 ± 0.15	0.62
Neu, 10^3^/μl, Mean ± SD	4.87 ± 0.09	4.61 ± 0.11	4.21 ± 0.19	0.01
Hb, g·dl, Mean ± SD	13.48 ± 0.10	14.31 ± 0.11	15.05 ± 0.21	< 0.0001
Plt, 10^3^/μl, Mean ± SD	236.34 ± 4.43	222.26 ± 4.25	220.09 ± 13.33	0.06
SBP, mmHg, Mean ± SD	130.40 ± 1.14	129.27 ± 1.61	127.41 ± 4.75	0.73
DBP, mmHg, Mean ± SD	65.62 ± 0.88	67.91 ± 1.01	71.97 ± 2.40	0.02

#### 3.6.2. The association between CHF with frailty and non-frailty

**Figure 7:**
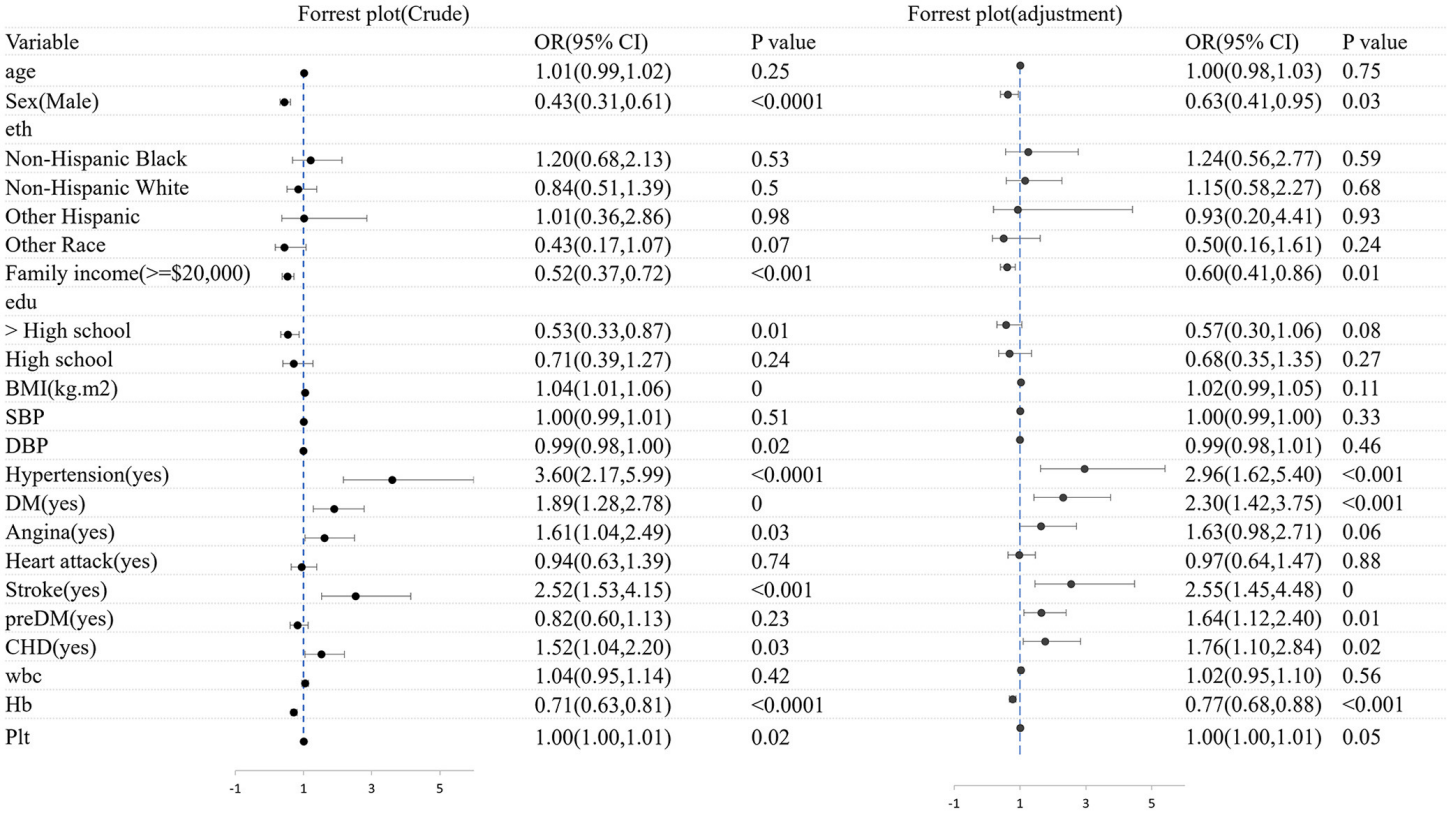
The logistic regression analysis of CHF patients with frailty and non-frailty before and after adjustments for the covariates.

Figure 7 shows the logistic regression analysis of CHF patients with frailty and non-frailty before and after adjustments for the covariates. We found that gender and family income were significantly associated with CHF patients with frailty (before and after adjustments, *P* < 0.05).

It was found that female patients were more likely to acquire CHF with frailty than male patients (OR = 0.43, 95% CI: 0.31–0.61). After adjusting factors of age, gender, ethnicity, family income, education, BMI, diseases, and blood test, the association was slightly weakened (OR = 0.63, 95% CI: 3.11–5.22). A statistically significant distinction was found between increased OR CHF with frailty and lower yearly family income (OR = 0.52, 95% CI: 0.37–0.72, *p* < 0.001). The OR was 0.06 after the factors of age, sex, ethnicity, family income, education, BMI, diseases, and blood test were adjusted. There was a significant association between lower yearly family income and the higher prevalence of CHF with frailty (OR = 0.06, 95 CI: 0.41–0.86, *P* < 0.01). Besides, CHF patients with frailty tended to suffer from hypertension (OR = 3.60, 95% CI: 2.17–5.99, *P* < 0.0001), stroke (OR = 2.52, 95% CI: 1.53–4.15, *P* < 0.001), DM (OR = 1.89, 95% CI: 1.28–2.78, *P* < 0.001), and CHD (OR = 1.52, 95% CI: 1.04–2.20, *P* < 0.001), respectively, than those without frailty. Except for hypertension (OR = 2.96, 95% CI: 1.62–5.40, *P* < 0.001), the rest variable of stroke was (OR = 2.55, 95% CI: 1.45–4.48, *P* < 0.001), DM was (OR = 2.30, 95% CI: 1.42–3.75, *P* < 0.001), and CHD was (OR = 1.76, 95% CI: 1.10–2.84, *P* = 0.02) after being adjusted. We also found that the Hb level improved by 1 g·dl, and the risk of CHF may decrease by 33% (OR = 0.77, 95% CI: 0.68–0.88, *P* < 0.001).

#### 3.6.3. The association between cardiac and cerebrovascular disease and CHF

We analyzed the correlation between CHD and CHF to identify protective and risk factors in the disease process. In the logistic regression analysis, frailty, a higher BMI, and co-morbidities (including DM, angina, heart attack, and stroke) are all risk factors for CHF. After adjusting factors of age, gender, ethnicity, family income, education, BMI, diseases, and blood test, frailty was still strongly associated with CHF (OR = 3.33, 95% CI: 2.21–5.01, *P* < 0.0001). Higher BMI (OR = 1.04, 95% CI: 1.01–1.07, *P* = 0.01), angina (OR = 1.50, 95% CI: 1.09–2.06, *P* = 0.01), heart attack (OR = 1.93, 95% CI: 1.36–2.73, *P* < 0.001), and stroke (OR = 1.73, 95% CI: 1.17–2.54, *P* = 0.01) were risk factors for CHF, and high school education or above was a protective factor for CHF (Supplementary Table S3).

For DM, frailty, advanced age, race (including Non-Hispanic black and Non-Hispanic white), high BMI, and co-morbidities (including hypertension, CHD, angina, heart attack, and stroke) were risk factors for CHF, while high income, high educational level, and elevated DBP were protective factors. Frailty (OR = 3.47, 95% CI: 2.47–4.86, *P* < 0.0001), advanced age (OR = 1.03, 95% CI: 1.01–1.05, *P* = 0.003), Non-Hispanic black (OR = 2.02, 95% CI: 1.25–3.27, *P* = 0.004), and co-morbidities (including hypertension, CHD, heart attack, and stroke) still exacerbated the likelihood of CHF after the correction for model (Supplementary Table S4).

The above-mentioned studies suggested that we should screen for frailty early in CHD and DM to prevent heart failure. Moreover, controlling weight could reduce the likelihood of CHF in CHD and DM. Patients with one or more cardiac and cerebrovascular diseases should measure frailty as soon as possible.

#### 3.6.4. The association between CHF with morbidity and FI

To further explore whether CHF comorbidities aggravate frailty, subgroup analyses were performed. As shown in Table 4, the FI of CHF patients with CHD was generally higher than those without CHD (0.33 ± 0.01 vs. 0.29 ± 0.1, *P* < 0.001). However, the increase in FI varies from different characteristics. Compared with female patients, male patients had a more significant increase in FI (*P* < 0.01 vs. *P* < 0.0001). Patients with CHF having less than high school education, with a BMI of 20–25, had a more significant increase in FI after CHD than the other patients.

**Table 4:** Subgroup analysis for CHF with CHD.

**Characteristic**	**CHF without CHD**		**CHF With CHD**		***P*-value**

	***n*** **(%)**	**FI (mean** ±**SD)**	***n*** **(%)**	**FI (mean** ±**SD)**	
Gender					< 0.0001
Female	319 (57.83)	0.32 ± 0.01	153 (36.23)	0.36 ± 0.01^**^	
Male	309 (42.17)	0.25 ± 0.01	344 (63.77)	0.31 ± 0.01^****^	
Race					0.06
Mexican American	57 (3.45)	0.31 ± 0.02	51 (2.96)	0.32 ± 0.02	
Non-hispanic black	184 (14.86)	0.31 ± 0.01	68 (6.99)	0.36 ± 0.02^***^	
Non-hispanic white	332 (74.00)	0.29 ± 0.01	311 (81.66)	0.33 ± 0.01^****^	
Other hispanic	35 (4.49)	0.35 ± 0.02	40 (4.68)	0.34 ± 0.03	
Other race	20 (3.20)	0.24 ± 0.03	27 (3.72)	0.30 ± 0.02	
Annual family income					0.27
< $20,000	268 (41.65)	0.32 ± 0.01	205 (37.07)	0.36 ± 0.01^***^	
≥$20,000	360 (58.35)	0.28 ± 0.01	292 (62.93)	0.31 ± 0.01^***^	
Education					0.3
< High school	109 (15.56)	0.32 ± 0.01	100 (12.77)	0.36 ± 0.02^**^	
>High school	229 (39.25)	0.28 ± 0.01	184 (36.55)	0.31 ± 0.01	
High school	290 (45.19)	0.29 ± 0.01	213 (50.68)	0.34 ± 0.01^****^	
BMI					0.68
≤ 20	20 (3.20)	0.25 ± 0.02	15 (2.24)	0.38 ± 0.02	
>30	335 (51.05)	0.31 ± 0.01	240 (51.20)	0.34 ± 0.01	
20–25	95 (16.35)	0.25 ± 0.01	79 (14.13)	0.33 ± 0.02^***^	
25–30	178 (29.41)	0.29 ± 0.01	163 (32.44)	0.32 ± 0.01	
FI Mean ± SD	0.29 ± 0.01		68.40 ± 0.86		0.53
WBC, 10^3^/μl, Mean ± SD	7.55 ± 0.11		7.85 ± 0.14		0.06
Plt, 10^3^/μl, Mean ± SD	240.21 ± 4.14		219.93 ± 4.24		< 0.001
BMI kg.m^2^	32.07 ± 0.58		30.98 ± 0.37		0.09

T-tests compare with CHF without DM, ^**^means P < 0.01; ^***^means P < 0.001; ^****^means P < 0.0001.

As shown in Table 5, FI increased after suffering from DM (0.29 ± 0.01 vs. 0.34 ± 0.01, *P* < 0.001). FI of male patients increased more significantly than that of female patients (*P* < 0.0001 vs. *P* < 0.01). The frailty of the BMI > 30 subgroup increased more significantly with DM (0.29 ± 0.01 vs. 0.35 ± 0.01, *P* < 0.001). FI of Mexican American and Non-Hispanic white increased after the DM combination. Overall, it is suggested that FI increased in CHF patients with CHD and DM.

**Table 5:** Subgroup analysis for CHF with DM.

**Characteristic**	**CHF without DM**		**CHF with DM**		***P*-value**

	***n*** **(%)**	**FI (mean** ±**SD)**	***n*** **(%)**	**FI (mean** ±**SD)**	
Gender					0.29
Female	243 (50.22)	0.32 ± 0.01	229 (45.65)	0.36 ± 0.01^**^	
Male	324 (49.78)	0.26 ± 0.01	329 (54.35)	0.32 ± 0.01^****^	
Race					0.05
Mexican American	49 (2.68)	0.26 ± 0.02	59 (3.97)	0.36 ± 0.02^***^	
Non-hispanic black	100 (8.96)	0.30 ± 0.01	152 (14.61)	0.34 ± 0.01	
Non-hispanic white	360 (79.76)	0.29 ± 0.01	283 (74.22)	0.34 ± 0.01^****^	
Other hispanic	31 (5.40)	0.33 ± 0.03	44 (3.45)	0.39 ± 0.02	
Other race—including multi-racial	27 (3.19)	0.28 ± 0.03	20 (3.75)	0.27 ± 0.02	
Annual family income					0.05
< $20,000	238 (42.82)	0.32 ± 0.01	235 (35.34)	0.36 ± 0.01^***^	
≥$20,000	329 (57.18)	0.27 ± 0.01	323 (64.66)	0.32 ± 0.01^****^	
Edu					0.08
< High school	89 (12.40)	0.32 ± 0.01	120 (16.90)	0.35 ± 0.02	
>High school	209 (36.22)	0.26 ± 0.01	204 (40.51)	0.33 ± 0.01^****^	
High school	269 (51.38)	0.30 ± 0.01	234 (42.59)	0.34 ± 0.01^**^	
BMI					< 0.0001
≤ 20	27 (4.09)	0.28 ± 0.02	8 (1.01)	0.36 ± 0.04	
>30	214 (40.33)	0.29 ± 0.01	361 (65.55)	0.35 ± 0.01^***^	
20–25	118 (20.70)	0.28 ± 0.01	56 (8.21)	0.30 ± 0.02	
25–30	208 (34.87)	0.29 ± 0.01	133 (25.23)	0.32 ± 0.01	
Age mean ± SD	67.82 ± 0.90		68.30 ± 0.53		0.63
FI mean ± SD	0.29 ± 0.01		0.34 ± 0.01		< 0.0001
WBC, 10^3^/μl, Mean ± SD	7.51 ± 0.13		7.91 ± 0.13		0.04
Plt, 10^3^/μl, Mean ± SD	237.42 ± 4.77		222.94 ± 3.81		0.02
BMI kg/m^2^	29.92 ± 0.57		33.82 ± 0.43		< 0.0001

T-tests compare with CHF without DM, ^**^means P < 0.01; ^***^means P < 0.001; ^****^means P < 0.0001.

### 3.7. Survival analysis for frailty and all-cause mortality and CVD mortality

In cardiovascular and cerebrovascular participants, altogether 4,912 deaths were documented including 1,376 CVD-related deaths. As shown in Table 6, after adjustment of gender, age, education, ethnicity, family income, BMI, WBC, Plt, Hb, and morbidity, pre-frailty and frailty remain a higher relationship between all-cause and CVD mortality. The multivariate-adjusted HRs and 95% CIs for all-cause mortality from non-frailty, pre-frailty, to frailty were 1.00 (reference), 1.48 (1.32, 1.66), and 2.77 (2.40, 3.18), respectively. For CVD mortality, non-frailty, pre-frailty, and frailty were 1.00 (reference), 1.58 (1.26, 1.97), and 2.55 (2.02, 3.21), respectively. This analysis suggested that for cardiovascular and cerebrovascular participants, frailty measurement was a convenient way to judge both all-cause and CVD survival and prognosis.

**Table 6:** All-cause mortality and CVD mortality in cardiovascular and cerebrovascular participants and CHF patients.

	**Crude**		**Adjusted**		

	***P*** **-values**	**HR (95%Cl)**	***P*** **-values**	**HR (95%Cl)**	**Deaths/total**
**All-cause mortality in cardiovascular and cerebrovascular illnesses**					
None	Ref	Ref	Ref	Ref	1,140/4,940
Pre-frailty	< 0.0001	1.54 (1.40, 1.69)	< 0.0001	1.48 (1.32, 1.66)	2,211/7,225
Frailty	< 0.0001	2.71 (2.41, 3.04)	< 0.0001	2.77 (2.40, 3.18)	1,561/3,994
**CVD mortality in cardiovascular and cerebrovascular illnesses**					
None	Ref	Ref	Ref	Ref	287/4,940
Pre-frailty	< 0.0001	1.87 (1.50, 2.35)	< 0.0001	1.58 (1.26, 1.97)	627/7,225
Frailty	< 0.0001	3.33 (2.74, 4.04)	< 0.0001	2.55 (2.02, 3.21)	462/3,994
**All-cause mortality in CHF patients**					
None	Ref	Ref	Ref	Ref	14/31
Pre-frailty	0.46	1.21 (0.73, 1.99)	0.31	1.31 (0.78, 2.21)	157/348
Frailty	0.02	1.82 (1.09, 3.03)	0.004	2.14 (1.27, 3.62)	422/745
**CVD mortality in CHF patients**					
None	Ref	Ref	Ref	Ref	5/31
Pre-frailty	0.54	1.33 (0.54, 3.25)	0.37	1.40 (0.67, 2.93)	60/348
Frailty	0.17	1.85 (0.76, 4.48)	0.06	2.10 (0.97, 4.53)	165/745

In 1,124 CHF patients with followed-up history, 593 deaths were recorded, including 230 CVD deaths. Compared with non-frailty CHF patients, the ratio of all-cause mortality in patients with frailty increased. The multivariate-adjusted HRs and 95% CIs for all-cause mortality from non-frailty to frailty are 1.00 (reference), 1.31 (0.78, 2.21), and 2.14 (1.27, 3.62), respectively. However, the ratio of CVD mortality increased without significance. The multivariate-adjusted HRs and 95% CIs for CVD mortality from non-frailty to frailty are 1.00 (reference), 1.40 (0.67, 2.93), and 2.10 (0.97, 4.53), respectively. This analysis suggested that CHF patients with frailty were associated with a poor all-cause survival rate and prognosis.

## 4. Discussion

To the best of our knowledge, our publication is the first NHANES-related study to assess the association between CHF and FI, taking into account both clinical and laboratory evaluations, which is significant for epidemiological research into CHF. Recently, the 2022 AHA/ACC/HFSA Guideline for the Management of Heart failure was published. Frailty was listed as a medical barrier to CHF self-care in stage C. Our study provides new evidence from the NHANES database that frailty is common in patients with CHF, and the FI can be used as a prognostic information both in patients with CHF and patients with cardiac and cerebrovascular disease. CHF patients with hypertension, DM, stroke, and CHD are positively connected with frailty while patients with CHF who are male, with annual family income over $20,000, and a higher Hb were protective factors to be frail. We also found that CHF patients with CHD or DM were frailer than patients without those two diseases. For patients with cardiovascular and cerebrovascular diseases and CHF, assessing frailty may be an effective way to predict the prognosis of patients.

### 4.1. Frailty measurement in CHF

Multiple frailty measurements have been created to screen and assess frailty (11, 12). We mainly used the scale to assess the degree of frailty, some of which are listed in Table 7. The phenotype model and the cumulative deficits model are the two main emerging models (13). The phenotype model, also known as Fried's phenotype, is widely used in CVD and is characterized by a short-time assessment. Five variables including unintentional weight loss, self-reported exhaustion, low energy expenditure, slow gait speed, and weak grip strength are contained in the model. Frailty is defined by those with more than two factors mentioned above (14). The Cumulative Deficit Model developed by Rockwood considers frailty as a clinical state of the accumulation of deficits. These deficits are combined in an index score to reflect the proportion of potential deficits present in the cumulative deficit model (9). The index is based on a comprehensive geriatric assessment and combines four parts. The model provides a predictor of poor health outcomes, which accounts for a cumulative explanation of frailty to some extent. The frailty index in the Cumulative Deficit Model is expressed as a ratio of a cumulative of all potential deficits of the index. The Edmonton frailty scale (EFS) is a multidimensional frailty assessment tool that includes general health status, functional independence, social support, cognition, medication use, nutrition, continence, and mood (15). Physical frailty was defined according to the results of two tests of physical abilities in Gill's study (16), which were strongly associated with the development and progression of disability. Moderately frail was defined as a rapid gait of >10 s or could not stand from the chair. Persons meeting both criteria were considered severely frail. Handgrip strength was obtained by hand grip dynamometer with usually multiple attempts allowed. The scores were based on the best performance or average performance. Although none of the scales seems to recognize a gold standard for the measurement and screening of frailty, the Heart Failure Association of the European Society of Cardiology emphasized that frailty could not be seen as a synonym for aging, physical limitations, and disease severity. Thus, the assessment of frailty should be performed with a multidimensional tool that should take into account psychological, social, and clinical factors, in addition to physical limitations. Based on the above, we chose the Cumulative Deficit Model to evaluate frailty in patients with heart failure.

**Table 7:** Comparison of different frailty scales.

**Scale**	**Strengths**	**Weaknesses**
Cumulative deficit model	Easy and fast to perform; a continuous scoring system including 3 domains. (Physical, psychological, and social)	Complex to use because of its mathematical nature
Fried frailty phenotype	Predictive of adverse clinical outcome	Including physical testing; conducted in stable, mobile patients
Edmonton frailty scale	Moderately complex multidimensional scale; an independent predictor of unscheduled re-hospitalization	Including physical testing; conducted in stable, mobile patients
Gill frailty measure	Quick, precise, objective measurement; highly correlated with other functional tests in HF	Sensitive but not specific for frailty by most common cutoffs; limited to ambulatory patients; Score may be affected by the type of chair and assistive devices
Handgrip strength	Rapid, objective measurement; no ambulation required, safety	Heterogeneity in testing protocols; measurement tools not universally available

### 4.2. Prevalence of frailty in CHF

Several studies have found a high frequency of frailty in people with CHF. Over 90% of patients in the Treatment of Preserved Cardiac Function Heart Failure with an Aldosterone Antagonist (TOPCAT) study were frail (FI = 0.37 ± 0.11) (17). Frailty was found in 79.4% of 811 persons over the age of 65 in another investigation using the Rockwood FI (5). Similar results were obtained in our study; 64.1% of patients with CHF were frailty, 33.22% of them were in a state of pre-frailty, and only 2.67% of participants showed non-frailty. Logistic regression suggested the risk of frailty in patients with CHF was 3.79 (OR = 3.79, 95% CI: 2.97–4.83) times higher than in patients with non-CHF.

### 4.3. Male–Female health-survival paradox

Female patients are frailer and have poorer health near the end of life, whereas male patients continue to outperform female patients on physical function tests, which is known as the sex paradox. In our study, female patients with CHF were more likely to be frail than male patients with CHF. Other studies indicated a greater heritability of several psychological and neurological features in female patients, such as depression (18), aches (19), fatigue, and insomnia, which was ascribed to the higher prevalence of these traits in female patients.

The results of a meta-analysis by Gordon et al. (20) including five studies suggested that female patients had greater frailty ratings than male patients in all age categories, while male patients' mortality was lower than female patients until the 90–94 years age range. Scientists have investigated the causes of the gender paradox. Female patients may be more prone to suffer non-fatal chronic illnesses, but male patients tend to get urgent disorders with a high mortality rate, such as stroke and myocardial infarction. Female patients are more active in seeking medical attention, while male patients usually tend to underestimate the morbidity and handicap of their diseases (21).

Recent studies have evaluated targeted interventions such as supervised exercise training to reduce the frailty burden and improve heart failure patient-reported and clinical outcomes (22, 23). It is an effective way for female patients to increase muscle strength before being frail. In addition, frailty and chronic diseases make the elderly have a higher incidence of negative psychological emotions (24), and female patients are more likely to be affected. Psychological support is necessary and important. Multiple studies have shown that low self-efficacy always results in a higher incidence of frailty, poor self-management, medication compliance, and quality of life (25, 26). The self-efficacy of patients, especially female patients, can be improved using goal setting, peer education, and incentive mechanism (27).

### 4.4. Normal Hb is the protective factor against CHF and frailty

According to a meta-analysis (27), more than 50% of the studies showed that frail individuals had lower levels of red blood cells, especially Hb, compared with non-frail adults. In our study, we drew a similar conclusion. Previous studies showed that low Hb and anemia might reduce tissue oxygenation, decrease muscle synthesis and strength, and lead to weakness (28, 29). In addition to qualitative research, there was also quantitative research. It showed that decreasing Hb was associated with increased comorbidity, frailty, and major adverse cardiovascular events (MACE). Hb could independently predict MACE, and 140 g/L was the optimal cutoff point for predicting MACE (30). Recent studies showed that intravenous ferric carboxymaltose was effective to improve CHF patients' health-related quality of life with anemia (31). Impaired iron homeostasis could be one mechanism underlying the poor physical condition of patients with CHF.

### 4.5. Higher family yearly income and high education are protective factors against CHF and frailty

A meta-analysis of 56 studies found that the prevalence of frailty among the elderly in high- and middle-income countries was 8.2 and 12.3%, respectively (32). We came to a similar conclusion. Moreover, we found that education was a protective factor for both CHF and frailty but was not so important in the association between CHF and frail. Another study investigating the twin-based heritability of frailty discovered a minimal genetic association between FI and education (33). The higher family income may contribute to higher education, which then imports health consciousness, assisting in the prevention of frailty development consequently (34, 35). Besides, the healthcare-seeking behavior was especially characteristic of female patients with high education.

These results suggested that in future, the healthcare system should provide universal awareness of the disease so that patients could be diagnosed and treated at an early stage. For low-income patients, the completion of the insurance system can guarantee effective and comprehensive medical care, which is another important prerequisite. Finally, more disease prediction models and detection tools will be invented with big data and new technologies. Early screening can be used to predict diseases and thus delay their onset. Disease education should be provided to poor patients so that they can be detected early and treated promptly, and reduce the cost of medical care.

### 4.6. Comparison of differences with other related studies

Several studies have been conducted on heart failure and frailty. A study conducted by Kitzman et al. found that 3-month physical rehabilitation improved cardiac function in patients with acute decompensated heart failure (ADHF) combined with frailty. At 6 months, the rates of rehospitalization for any cause in the intervention were also reduced. However, the death rate from any cause did not decrease (36). Dapagliflozin and Prevention of Adverse Outcomes in Heart Failure suggested that dapagliflozin could improve all outcomes examined regardless of frailty status, and the prevalence of frailty was 53.19% (37). In the TOPCAT trial (17), FI increased with a higher BMI, systolic blood pressure, and pulse pressure. The mean age was lowest for the frailest class. The prevalence of frailty was about 94%. Our study produced a similar result to the above study. Frailty is closely related to heart failure. While most of the above studies were randomized controlled trials, these limited the participants (ADHF and heart failure with preserved ejection fraction patients) or excluded the frailest or the least frail patients. Our publication was a study based on the NHANES with a long follow-up time and complete data information. Patients included most types of heart failure. The gender distribution of patients was even. Second, no correlation was found among BMI, SBP, and pulse pressure in the subgroup of patients with CHF in our study. The reason may be related to the fact that most types of patients with CHF were included. However, our result suggested that patients with CHF who were male, had annual family income over $20,000, or with normal Hb were protective factors against frailty. Third, we found that for both patients with cardiac and cerebrovascular disease and patients with CHF, frailty was strongly connected with an increased hazard of all-cause death.

### 4.7. Limitations

The following are the limitations of this study. First, as a cross-sectional study, it was not possible to draw a cause-and-effect conclusion between frailty and CHF yet. Second, limited to the diagnosis of the NHANES database and the laboratory data, we cannot perform subgroup analysis of ischemic heart failure and sarcopenia. We also cannot analyze the association between Brain Natriuretic Peptide (BNP) and frailty as well as the cardiac ultrasound data. Third, because the participants reported the prevalent ailments themselves, there may be bias due to recollection. Finally, our study was restricted by database constraints to the US individuals only, so there could be geographical constraints, which meant our findings could not be generalized to all patients worldwide.

### 4.8. Future directions

The main implications of our research for future are as follows. First, the risk of frailty in patients with CHF is over three times higher than in patients with non-CHF according to our research. And a great proportion of cardiac and cerebrovascular disease and other chronic diseases were suffering from frailty, including hypertension, angina, coronary heart disease, stroke, and diabetes mellitus. Doctors need to focus on the management of frail patients with CHF in future studies. Additionally, gender-based studies of frailty in CHF could be undertaken. Third, it was found that family income played an important role in the risk of frailty. The awareness of frailty should be spread among patients with CHF, especially in remote and underdeveloped areas around the world. Finally, more programs could be explored according to different etiologies, Ejection fraction, and type B natriuretic peptide, to identify the risk factors of frailty over the world.

## 5. Conclusion

In conclusion, our analysis revealed that frailty was strongly associated with CHF. Patients with CHF who were male, had annual family income over $20,000, or with normal Hb level were negatively correlated to frailty. For both patients with cardiac and cerebrovascular disease and patients with CHF, frailty was strongly connected with an increased hazard of all-cause death.

## Data availability statement

The original contributions presented in the study are included in the article/Supplementary material, further inquiries can be directed to the corresponding author.

## Ethics statement

Ethical review and approval were not required for the study on human participants in accordance with the local legislation and institutional requirements. Written informed consent for participation was not required for this study in accordance with national legislation and institutional requirements.

## Author contributions

XC, BL, CH, and XZ conceived the ideas and design of the study. XC and CH collected data from NHANES. YM, LY, JL, and XZ analyzed the data. MG, YL, and XC drafted the manuscript. DF and MW revised the final version of the manuscript and supervised the study. XC and CH contributed equally to this study and share the first authorship. All authors have read and approved the final version of the manuscript for publication.
