# Supplementary material for: The association between chronic heart failure and frailty index: A study based on the National Health and Nutrition Examination Survey from 1999 to 2018
Source: Front Cardiovasc Med. 2023 Jan 9;9:1057587. doi: 10.3389/fcvm.2022.1057587 (PMC9868664; doi:10.3389/fcvm.2022.1057587)
Supplement: Supplementary file 1 [file Table_1.DOCX]

**Supplementary Table**

Table S1 Variables in the 53-Item Frailty Index and Their Respective Scorings

| Variable | Scoring |
| --- | --- |
| Cognition |  |
| 1.Experience confusion memory problems | Yes=1, No=0 |
| Dependence |  |
| 2.Money manage | Difficulty=1, No Difficulty=0 |
| 3.Walking for a quarter mile | Difficulty=1, No Difficulty=0 |
| 4.Walking up ten steps | Difficulty=1, No Difficulty=0 |
| 5.Stooping, crouching, kneeling | Difficulty=1, No Difficulty=0 |
| 6.Lifting or carrying | Difficulty=1, No Difficulty=0 |
| 7.House chore | Difficulty=1, No Difficulty=0 |
| 8.Preparing meals | Difficulty=1, No Difficulty=0 |
| 9.Walking between rooms on same floor | Difficulty=1, No Difficulty=0 |
| 10.Standing up from armless chair | Difficulty=1, No Difficulty=0 |
| 11.Getting in and out of bed | Difficulty=1, No Difficulty=0 |
| 12.Using fork, knife, drinking from cup | Difficulty=1, No Difficulty=0 |
| 13.Dressing yourself | Difficulty=1, No Difficulty=0 |
| 14.Standing for long periods | Difficulty=1, No Difficulty=0 |
| 15.Sitting for long periods | Difficulty=1, No Difficulty=0 |
| 16.Reaching up over head | Difficulty=1, No Difficulty=0 |
| 17.Grasp/holding small objects | Difficulty=1, No Difficulty=0 |
| 18.Going out to movies events difficulty | Difficulty=1, No Difficulty=0 |
| 19.Attending social event | Difficulty=1, No Difficulty=0 |
| 20.Leisure activity at home | Difficulty=1, No Difficulty=0 |
| 21.Push or pull large objects | Difficulty=1, No Difficulty=0 |
| Depressive Symptoms |  |
| 22.Have little interest in doing things | Nearly every day=1, More than half the days=0.66, Several days=0.33, Not at all=0 |
| 23.Feeling down, depressed, or hopeless | Nearly every day=1, More than half the days=0.66, Several days=0.33, Not at all=0 |
| 24.Trouble sleeping or sleeping too much | Nearly every day=1, More than half the days=0.66, Several days=0.33, Not at all=0 |
| 25.Feeling tired or having little energy | Nearly every day=1, More than half the days=0.66, Several days=0.33, Not at all=0 |
| 26.Poor appetite or overeating | Nearly every day=1, More than half the days=0.66, Several days=0.33, Not at all=0 |
| 27.Feeling bad about yourself | Nearly every day=1, More than half the days=0.66, Several days=0.33, Not at all=0 |
| 28.Trouble concentrating on things | Nearly every day=1, More than half the days=0.66, Several days=0.33, Not at all=0 |
| Comorbidities |  |
| 29.Aarthritis | Yes=1, Suspect=0.5, No=0 |
| 30.Thyroid problems | Yes=1, Suspect=0.5, No=0 |
| 31.Chronic bronchitis | Yes=1, Suspect=0.5, No=0 |
| 32.Cancer | Yes=1, Suspect=0.5, No=0 |
| 33.Congestive heart failure | Yes=1, Suspect=0.5, No=0 |
| 34.Coronary heart disease | Yes=1, Suspect=0.5, No=0 |
| 35.Angina | Yes=1, Suspect=0.5, No=0 |
| 36.Heart attack | Yes=1, Suspect=0.5, No=0 |
| 37.Stroke | Yes=1, Suspect=0.5, No=0 |
| 38.High blood pressure | Yes=1, Suspect=0.5, No=0 |
| 39.Diabetes | Yes=1, Suspect=0.5, No=0 |
| 40.Weak/failing kidneys | Yes=1, Suspect=0.5, No=0 |
| 41.Urinary leakage | Yes=1, Suspect=0.5, No=0 |
| Hospital Utilization and Access to Care |  |
| 42.General health condition | Fair, poor=1, Excellent, Very good, good=0 |
| 43.Number of prescribed medications | None=0, 1-4=0.5, 5 and more=1 |
| 44.Overnight hospital patient | Yes=1, No=0 |
| 45.Health compared 1 year ago | Worse=1, About the same, Better=0 |
| 46.Times receive healthcare over past year | None=0, 1-5=0.5, More than 5=1 |
| Physical Performance and Anthropometry |  |
| 47.Body mass index | <18.5, ≥30=1  25-<30=0.5  18.5-25=0 |
| Laboratory Values |  |
| 48.Glycohemoglobin(%) | 0-5.7=0, >5.7=1 |
| 49.Red blood cell count (million cells/mL) | M: 4.7-6.1=0, Other=1; F: 4.2-5.4=0, Other=1 |
| 50.Hemoglobin (g/dL) | M:13.5-18=0, Other=1; F: 12-16=0, Other=1 |
| 51.Red cell distribution width (%) | 11.6-14.6=0, Other=1 |
| 52.Lymphocyte percent(%) | 20-40=0, Other=1 |
| 53.Segmented neutrophils percent(%) | 40-80=0, Other=1 |

Table S2 Co-linearity study of possible co-linear relationships between different factors

|  | | | Beta | t | P value | VIF |
| --- | --- | --- | --- | --- | --- | --- |
| Characteristics |  |  |  |  |  |  |
| Age | 0.001 | 0.000 | 0.049 | 5.558 | 0.000 | 1.573 |
| Sex | 0.011 | 0.004 | 0.022 | 2.657 | 0.008 | 1.369 |
| Mexican American | -0.008 | 0.006 | -0.011 | -1.262 | 0.207 | 1.611 |
| Non-Hispanic Black | 0.010 | 0.006 | 0.017 | 1.873 | 0.061 | 1.628 |
| Other Hispanic | -0.004 | 0.007 | -0.004 | -0.569 | 0.570 | 1.229 |
| Other Race or Multi-Racial | -0.012 | 0.008 | -0.012 | -1.611 | 0.107 | 1.166 |
| Annual family income | -0.001 | 0.004 | -0.002 | -0.326 | 0.744 | 1.146 |
| Edu | 0.003 | 0.006 | 0.005 | 0.557 | 0.577 | 1.509 |
| BMI | 0.001 | 0.000 | 0.034 | 4.476 | 0.000 | 1.208 |
| SBP | 0.000 | 0.000 | -0.027 | -3.189 | 0.001 | 1.475 |
| DBP | 8.890E-05 | 0.000 | 0.005 | 0.550 | 0.582 | 1.437 |
| Hypertension | 0.013 | 0.005 | 0.021 | 2.550 | 0.011 | 1.322 |
| DM | 0.007 | 0.005 | 0.013 | 1.377 | 0.169 | 1.746 |
| Angina | 0.063 | 0.008 | 0.061 | 7.895 | 0.000 | 1.214 |
| Heart.attack | 0.153 | 0.007 | 0.180 | 22.018 | 0.000 | 1.357 |
| Stroke | 0.019 | 0.007 | 0.021 | 2.819 | 0.005 | 1.092 |
| PreDM | 0.004 | 0.005 | 0.007 | 0.752 | 0.452 | 1.632 |
| Coronary heart diseases | 0.131 | 0.007 | 0.153 | 18.422 | 0.000 | 1.404 |
| Hb | -0.004 | 0.001 | -0.025 | -2.877 | 0.004 | 1.476 |
| PLT | 0.000 | 0.000 | -0.043 | -5.600 | 0.000 | 1.222 |
| Pre-frailty | -0.046 | 0.005 | -0.090 | -8.356 | 0.000 | 2.348 |
| Frailty | -0.043 | 0.011 | -0.073 | -3.992 | 0.000 | 6.875 |
| Frailty score | 0.569 | 0.039 | 0.242 | 14.625 | 0.000 | 5.590 |
| WBC | 0.013 | 0.006 | 0.211 | 2.206 | 0.027 | 185.690 |
| Lym | -0.014 | 0.006 | -0.186 | -2.399 | 0.016 | 122.808 |
| Neu | -0.010 | 0.006 | -0.072 | -1.585 | 0.113 | 41.802 |

Table S3 The association between CHD and CHF

| character | Crude | | Adjusted | |
| --- | --- | --- | --- | --- |
|  | P value | OR(95% CI) | P value | OR(95% CI) |
| Frailty (Frailty) | <0.0001 | 4.41(3.05,6.38) | <0.0001 | 3.33(2.21,5.01) |
| Age | 0.566 | 0.99(0.98,1.01) | 0.14 | 1.01(1.00,1.04) |
| Sex (Male) | 0.519 | 0.90(0.66,1.23) | 0.34 | 1.20(0.82,1.76) |
| eth |  |  |  |  |
| Mexican American | ref | ref | ref | ref |
| Non-Hispanic Black | 0.805 | 1.06(0.66,1.69) | 0.42 | 1.26(0.71,2.23) |
| Non-Hispanic White | 0.223 | 0.80(0.55,1.15) | 0.95 | 1.02(0.61,1.68) |
| Other Hispanic | 0.067 | 2.47(0.94,6.50) | 0.02 | 2.97(1.18,7.49) |
| Other Race | 0.092 | 0.57(0.30,1.10) | 0.44 | 0.74(0.34,1.60) |
| Family income(>=$20,00) | <0.0001 | 0.50(0.370,0.673) | 0.25 | 0.81(0.57,1.16) |
| edu |  |  |  |  |
| <High school | ref | ref | ref | ref |
| > High school | <0.0001 | 0.41(0.263,0.632) | 0.01 | 0.54(0.33,0.88) |
| High school | 0.15 | 0.72(0.464,1.126) | 0.31 | 0.76(0.45,1.29) |
| BMI (kg/m^2^) | <0.001 | 1.05(1.020,1.073) | 0.01 | 1.04(1.01,1.07) |
| SBP (mmHg) | 0.027 | 0.99(0.984,0.999) | 0.01 | 0.99(0.98,1.00) |
| DBP (mmHg) | 0.499 | 0.99(0.984,1.008) | 0.62 | 1.00(0.99,1.02) |
| Hypertension(yes) | 0.03 | 1.61(1.047,2.466) | 0.64 | 1.13(0.67,1.92) |
| DM (yes) | 0.003 | 1.53(1.162,2.008) | 0.5 | 1.15(0.77,1.72) |
| angina(yes) | <0.0001 | 2.01(1.531,2.630) | 0.01 | 1.50(1.09,2.06) |
| heart attack(yes) | <0.0001 | 2.29(1.666,3.143) | <0.001 | 1.93(1.36,2.73) |
| stroke(yes) | <0.0001 | 2.56(1.781,3.672) | 0.01 | 1.73(1.17,2.54) |
| Wbc(10^3^/μl) | 0.331 | 1.03(0.968,1.100) | 0.41 | 1.02(0.97,1.07) |
| Hb(g·dl) | 0.007 | 0.88(0.802,0.966) | 0.28 | 0.94(0.85,1.05) |
| Plt(10^3^/μl) | 0.45 | 0.99(0.997,1.001) | 0.08 | 1.00(1.00,1.00) |

Table S4 The association between DM and CHF

| character | Crude | | Adjusted | |
| --- | --- | --- | --- | --- |
|  | P value | 95% CI | P value | 95% CI |
| Frailty (Frailty) | <0.0001 | 6.06(4.60,7.97) | <0.0001 | 3.47(2.47,4.86) |
| age | <0.0001 | 1.03(1.02,1.04) | 0.003 | 1.03(1.01,1.05) |
| sex(Male) | 0.09 | 1.22(0.97,1.52) | 0.35 | 1.16(0.84,1.61) |
| eth |  |  |  |  |
| Mexican American | ref | ref | ref | ref |
| Non-Hispanic Black | 0.003 | 1.84(1.23,2.75) | 0.004 | 2.02(1.25,3.27) |
| Non-Hispanic White | <0.001 | 1.81(1.29,2.55) | 0.05 | 1.51(1.00,2.28) |
| Other Hispanic | 0.6 | 1.17(0.65,2.09) | 0.41 | 1.33(0.68,2.59) |
| Other Race | 0.7 | 0.86(0.39,1.90) | 0.97 | 1.02(0.37,2.85) |
| Family income(>=$20,00) | 0.02 | 0.74(0.57,0.95) | 0.29 | 0.85(0.63,1.15) |
| edu |  |  |  |  |
| <High school | ref | ref | ref | ref |
| > High school | 0.01 | 0.66(0.49,0.89) | 0.15 | 0.76(0.52,1.11) |
| High school | 0.09 | 0.75(0.54,1.05) | 0.06 | 0.67(0.44,1.02) |
| BMI (kg.m2) | <0.0001 | 1.03(1.02,1.05) | <0.0001 | 1.04(1.02,1.06) |
| SBP (mmHg) | 0.37 | 1.00(0.99,1.00) | 0.08 | 0.99(0.98,1.00) |
| DBP (mmHg) | <0.001 | 0.98(0.97,0.99) | 0.93 | 1.00(0.98,1.01) |
| Hypertension(yes) | <0.0001 | 3.38(2.37,4.83) | <0.001 | 2.13(1.40,3.26) |
| CHD (yes) | <0.0001 | 7.84(6.00,10.26) | <0.0001 | 2.74(1.85,4.06) |
| angina(yes) | <0.0001 | 4.97(3.74,6.61) | 0.26 | 1.24(0.85,1.82) |
| heart attack(yes) | <0.0001 | 8.88(6.84,11.52) | <0.0001 | 3.45(2.42,4.92) |
| Stroke (yes) | <0.0001 | 3.16(2.40,4.15) | 0.03 | 1.54(1.04,2.29) |
| Wbc(10^3^/μl) | 0.07 | 1.04(1.00,1.09) | 0.06 | 1.06(1.00,1.12) |
| Hb(g·dl) | <0.0001 | 0.85(0.79,0.91) | 0.21 | 0.93(0.84,1.04) |
| Plt(10^3^/μl) | <0.0001 | 1.00(0.99,1.00) | <0.001 | 1.00(0.99,1.00) |
